# The Impact of Biodex Balance on Improving Coordination and Static Balance in Patients with Diabetic Peripheral Neuropathy

**DOI:** 10.3390/life15071030

**Published:** 2025-06-27

**Authors:** Kristo Xhardo, Elona Xhardo, Mircea Bratu, Alin Pandea, Mariana Cordun, Ana-Maria Vasiliu, Dan-Eugen Costin, George Sebastian Iacob, Marjan Mihajlov, Ilie Onu, Daniel-Andrei Iordan

**Affiliations:** 1 Physiotherapie STAMP-Solingen Ohligs, 42697 Solingen, Germany; kristoxhardo@yahoo.com; 2 Department of Internal Medicine, “Shefqet Ndroqi” University Hospital, 1030 Tirana, Albania; xhardo.elona@gmail.com; 3 Faculty of Physical Therapy, National University of Physical Education and Sport, 060057 Bucharest, Romania; mircea.bratu@yahoo.com (M.B.); mariana_cordun@dr.com (M.C.); ana_vasiliu85@yahoo.com (A.-M.V.); 4 Faculty of Physical Education and Sport, “Dunarea de Jos” University, 800008 Galati, Romania; dan.costin@yahoo.com; 5 Department of Physical Therapy, Physiozentrum Däniken, 4658 Däniken, Switzerland; georgesebastianiacob@gmail.com; 6 Clinical Emergency Hospital, 105402 Bucharest, Romania; macoemk@yahoo.com; 7 Department of Biomedical Sciences, Faculty of Medical Bioengineering, University of Medicine and Pharmacy “Grigore T. Popa” Iasi, 700454 Iasi, Romania; 8 Center of Physical Therapy and Rehabilitation, “Dunărea de Jos” University of Galati, 800008 Galati, Romania; daniel.iordan@ugal.ro; 9 Department of Individual Sports and Kinetotherapy, Faculty of Physical Education and Sport, “Dunarea de Jos” University of Galati, 800008 Galati, Romania

**Keywords:** diabetic neuropathy, Biodex Balance System, balance training, proprioception, postural control, static balance

## Abstract

Diabetic peripheral neuropathy (DPN), a common complication of type 2 diabetes mellitus (T2DM), significantly impairs postural control and increases fall risk due to sensory and motor nerve dysfunction. While conventional rehabilitation is widely used, the effectiveness of technology-assisted balance training remains underexplored. This quasi-experimental study aimed to compare the impact of Biodex Balance System (BBS)-based training versus traditional exercises on balance and coordination in patients with DPN. Thirty patients with T2DM and clinically confirmed DPN were allocated into two groups (*n* = 15 per group): the intervention group (BBS training) and the control group (traditional exercises). Both groups trained for 8 weeks. Static balance was assessed using stability indices and clinical balance tests. Statistical analysis included paired and independent *t*-tests, Shapiro–Wilk tests for normality, and Cohen’s d for effect size. The BBS group demonstrated statistically significant improvements across all balance measures compared to the control group. For the most challenging condition (unstable surface, eyes closed), the mean balance index improved by 0.66° (*p* < 0.001; Cohen’s d = 14.25). Substantial improvements were also observed for the stable surface (eyes open: Δ = 0.34°, *p* < 0.001, d = 4.01) and unstable surface (eyes open: Δ = 0.23°, *p* < 0.001, d = 7.46). Control group gains were modest and less consistent. Balance training using the Biodex Balance System significantly enhances static balance and postural control in patients with diabetic neuropathy, outperforming traditional rehabilitation methods. These findings support integrating the BBS into structured diabetic care programs to reduce fall risk and improve functional stability.

## 1. Introduction

The literature published after 2021 and indexed in the Web of Science (WoS) shows an increased interest in topics such as diabetic peripheral neuropathy (DPN), the Biodex Balance System (BBS), and postural stability, with a significant volume of papers (over 3400 articles) reflecting the importance of these topics in the context of functional recovery in patients with type 2 diabetes mellitus (T2DM). We selected 335 articles based on the maximum co-occurrence of keywords in the texts. In the final stage, only 25 articles were chosen as having the most interesting non-repetitive content, most of them representing original research [1,2,3,4,5,6,7,8]. DPN affects nearly 50% of patients with long-standing T2DM, particularly involving cutaneous nerves such as the sural nerve [9].

DPN is a prevalent complication in patients with long-standing T2DM, affecting approximately 50% of this population. It predominantly impairs cutaneous nerves, such as the sural nerve, leading to deficits in proprioception and dynamic balance, and substantially increasing the risk of falls and mobility limitations [9]. The progressive degeneration of sensory and motor neurons further compromises postural stability, underscoring the clinical importance of targeted rehabilitation strategies [10].

Evidence from recent studies supports the effectiveness of BBS-based training in improving postural stability and reducing fall risk among individuals with DPN. Training with the BBS has been associated with improved outcomes on clinical assessments such as the Berg Balance Scale and the Timed Up and Go (TUG) test, indicating enhancements in both balance and coordination. The research domain addressing DPN and balance training is characterized by a strong interdisciplinary structure, with central themes including clinical evaluation, technology-assisted rehabilitation, biomechanical influences, cognitive–motor interactions, and the reliability of measurement tools [10,11,12].

Reyhanioglu et al. (2024) demonstrated that BBS training led to significant improvements in postural stability and reductions in fall risk and neuropathic pain, though no notable changes in nerve conduction were observed [13]. Similarly, Alaee et al. (2023) reported that hard-textured insoles improved balance in patients with diabetic neuropathy by enhancing sensory feedback [14]. The risk of falls is notably elevated in elderly individuals with DPN, with a two- to three-fold increase compared to non-neuropathic counterparts [15]. Additional research by Daud et al. (2021) and Eftekhar-Sadat et al. (2015) corroborated the benefits of BBS training for enhancing postural control in diabetic patients [10,16].

Recent developments also highlight the potential of computer-based and virtual feedback interventions in improving balance and motor coordination among individuals with neuropathy. For example, Reyhanioglu et al. (2024) found that balance exercises integrating virtual feedback improved postural responses and weight distribution, contributing to fall risk reduction [13]. These outcomes align with findings from Stolarczyk et al. (2021) [11], who evaluated proprioception, balance, muscle coordination, and strength in individuals with T2DM. Their study revealed significant impairments in the general stability index (GSI), frontal–posterior index (FPI), medial–lateral index (MLI), and fall risk in diabetic patients compared to healthy controls. Biofeedback training via the BBS led to significant improvements in these indices and a concomitant reduction in fall risk. The study also identified an age-dependent decline in static balance and a higher fall risk associated with increased body mass index (BMI) [11].

Orthotic interventions and sensory feedback tools represent an additional dimension of DPN rehabilitation. Given that DPN leads to diminished proprioceptive input from the lower limbs, the use of textured insoles and orthoses has demonstrated potential in enhancing sensory feedback. Alaee et al. (2023) documented immediate postural improvements with the use of textured insoles, supporting their integration into comprehensive rehabilitation programs alongside BBS training [14]. Stolarczyk et al. (2021) and Daud et al. (2021) also reported improvements in coordination and balance following BBS biofeedback training [10,11]. However, a study by Marchant et al. (2024) found that while compression-tactile socks improved somatosensory acuity, they did not significantly influence postural stability compared to barefoot conditions, possibly due to redundant feedback or insufficient postural challenge during the sensory organization test (SOT) [17].

Postural and motor control deficits in patients with DPN are often linked to neuromuscular dysfunction and proprioceptive deterioration. Motor nerve damage can contribute to muscle weakness, joint deformities, and altered gait mechanics, including conditions such as foot drop and Charcot foot, which may necessitate the use of assistive devices [8]. Stolarczyk et al. (2021) emphasized that BBS training is beneficial for both static and dynamic stability in elderly diabetic patients, recommending resistance and proprioceptive training as integral components of long-term rehabilitation strategies [11,18].

Postural control depends on the integration of visual, vestibular, and somatosensory systems. For instance, Nouraeinejad (2023) reported that childhood conditions such as strabismus and amblyopia could negatively impact adult postural stability, thereby highlighting the importance of binocular vision for balance maintenance [19]. Wafa et al. (2023) suggested that theoretical limits of stability (tLOS) may overestimate actual postural capabilities, as healthy adults demonstrated lower peak sway amplitudes (pLOSs), underscoring the need to refine balance assessment protocols [20].

Cognitive function and mental fatigue also play a role in postural control. Salehi et al. (2023) found that mental fatigue induced by cognitive tasks adversely affected balance in older adults [21]. Similarly, Loyd et al. (2021) examined gaze and balance deficits in people with multiple sclerosis using the WHO ICF framework [22]. Sariyeva et al. (2024) found that contact lenses provided better postural balance than spectacles in young individuals with myopia [23]. Salihu et al. (2022), using a dual-task paradigm, concluded that task difficulty and age had a more substantial impact on postural control than the complexity of the cognitive task itself [24]. Similarly, Saurabh Kumar and Gupta (2023) reported that cognitive function correlated with balance performance only in individuals with normal cognitive status; mild cognitive impairment did not yield significant differences [25].

Innovative technologies have been introduced to assess postural stability, including wearable sensors and video game-based force platforms. Aliperti et al. (2024) utilized such tools to distinguish between stable and unstable postural conditions [26]. Furthermore, Min et al. (2024) examined ergonomic influences on balance, finding that floor-sitting and kneeling postures reduced balance loss and muscle fatigue in an Asian sample, indicating the need for further research into posture-specific rehabilitation strategies [27].

In summary, the current body of evidence underscores the therapeutic value of BBS training, computer-assisted exercises, and orthotic devices in the rehabilitation of patients with DPN. These modalities have shown promise in enhancing postural stability, proprioceptive accuracy, and fall prevention. However, further investigation is warranted to address long-term sustainability, cost-effectiveness, and the impact on quality of life. Psychological comorbidities such as depression and anxiety frequently accompany DPN and may impede self-management. Therefore, early and comprehensive interventions are essential to improve functional outcomes, enhance independence, and mitigate suffering [15].

## 2. Materials and Methods

### 2.1. Study Design

This quasi-experimental study was designed to assess the effectiveness of balance training with the BBS in improving the postural control and coordination of patients with T2DM and DPN. The study was conducted over 10 months (April 2024 to January 2025) at the MEDIAN Heinrich-Mann-Klinik in Bad Liebenstein (Germany) and the Shefqet Ndroqi Tirana Hospital Center. It was structured into three phases:

Phase 1: Baseline assessment (April–November 2024). (a) Participants underwent initial balance and proprioception assessments using the Biodex Balance System and clinical sensory integration tests. (b) Demographic and medical data were recorded, including diabetes duration, neuropathy severity, and physical activity levels.

For the assessment, the patient is required to stand upright with their lower limbs positioned on the platform in areas D6 (left foot) and D16 (right foot), with a 10-degree angle between their calcaneus and hallux. Depending on the patient’s height, the support base can be adjusted.

Test steps: The patient must maintain a sway index as low as possible for 30 s while keeping their eyes open, with the platform static and no unstable surface. The patient must maintain a sway index as low as possible for 30 s while keeping their eyes closed, with the platform static and no unstable surface. The patient must maintain a balance index as low as possible for 30 s while keeping their eyes open, with the platform static, using an unstable surface. The patient must maintain a balance index as low as possible for 30 s while keeping their eyes closed, with the platform static, using an unstable surface.

Phase 2: Intervention (April–December 2024). The intervention lasted 8 weeks, with four supervised training sessions per week. Participants were allocated into two groups: experimental group (n = 15), participating in a personalized Biodex Balance training program; control group (n = 15), performing traditional balance and proprioceptive exercises without Biodex equipment.

Phase 3: Post-intervention assessment (December 2024–February 2025). The same balance and proprioception tests used in Phase 1 were conducted to assess improvements. Ultimately, the data were statistically analyzed to compare the effects of both interventions.

The study was conducted according to the Declaration of Helsinki and was approved by the Ethics Commission of the National University of Physical Education and Sport in Bucharest (Approval no. 33/27.09.2021). Written informed consent was obtained from all participants. Participants were free to withdraw at any stage without consequences.

### 2.2. Participants

A total of 30 patients diagnosed with T2DM and DPN were recruited from the Provita medical and the “Shefqet Ndroqi” Tirana Hospital Center. They were assigned to either the experimental or control group.

Inclusion criteria: (a) diagnosed with T2DM for at least 5 years; (b) the presence of DPN (confirmed via clinical examination); (c) age between 55 and 65 years; (d) able to walk independently, with or without assistive devices.

Exclusion criteria: (a) the presence of severe musculoskeletal disorders (e.g., foot ulcers fractures, joint replacements, degenerative joint disease); (b) a history of neurological conditions (e.g., stroke, Parkinson’s disease); (c) severe uncontrolled diabetes (HbA1c > 10%); (d) visual or vestibular impairments affecting balance; (e) the use of medications significantly impacting postural control (e.g., sedatives).

### 2.3. Intervention Protocol

Experimental group (Biodex Balance System SD, Biodex Medical Systems Inc., US): Participants performed balance and proprioception exercises using the Biodex Balance System. The training protocol included progressive difficulty levels targeting static and dynamic balance, proprioception, and weight shifting. The sessions lasted 20 min each and were conducted twice a day, totaling 16 sessions per month (BBS training).

Control group: Participants engaged in conventional balance exercises, including static standing balance (eyes open/closed), unilateral stance exercises, stepping and weight-shifting drills, therapeutic stretching, and lower-limb muscle strengthening. The duration and frequency of training were similar to those used for the experimental group.

### 2.4. Outcome Measures

They were related to balance and proprioception and were assessed using both instrumental and clinical methods:

− BBS Tests: stability index (measuring postural sway in degrees), sensory integration tests (assessing balance with eyes open/closed), and weight distribution analysis;
− Clinical Balance Tests: unstable surface balance test (eyes open/closed), Timed Up and Go Test, and Functional Reach Test;
− Statistical Analysis: descriptive statistics (mean, standard deviation), Shapiro–Wilk normality test, Levene’s test for homogeneity of variances, paired *t*-tests/Welch’s *t*-tests for pre- and post-intervention comparisons, and effect size (Cohen’s d) to assess clinical significance.

### 2.5. Statistical Analysis

Descriptive statistics (mean ± SD) were calculated for all outcome variables. Normality was assessed using the Shapiro–Wilk test. Homogeneity of variances was tested via Levene’s test. The tests depended on these assumptions:

- Parametric tests: Independent samples *t*-test (Student or Welch) for between-group comparisons.
- Nonparametric alternatives were applied where assumptions were violated.
- Effect sizes (Cohen’s d) were reported to assess the magnitude of intervention effects (interpreted as small = 0.2, medium = 0.5, large > 0.8).

A significance level of *p* < 0.05 was considered statistically significant. Statistical analyses were conducted using standard statistical software (**IBM SPSS Statistics vers 25)**.

## 3. Results

As can be seen in Table 1, the standard deviation is very small for all variables, meaning that the groups are homogenous. Our variable has a normal distribution, emphasizing a Shapiro–Wilk W-value greater than 0.9 and a high level of significance, with a *p*-value greater than 0.05. Exceptions: stable surface, eyes closed—final values (SS_closedF); unstable surface, eyes open/closed—final values (USS_openF/USS_closedF).

Age: Patients in both groups are of a similar age, with an average of about 60 years old.

### 3.1. Stable Surface—Eyes Open

**Figure 1:**
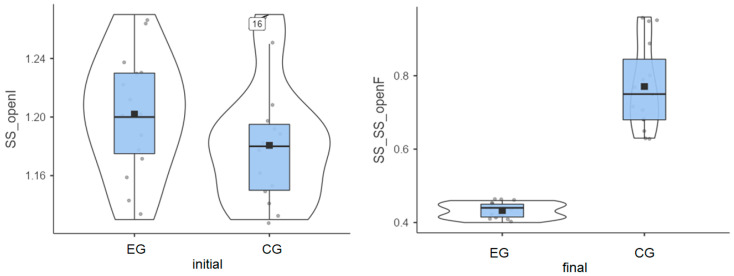
Stable surface (SS) distribution—eyes open: (**left**) initial values and (**right**) final values for experimental group (EG) and control group (CG).

Experimental group: The initial values measured for the 15 patients had an average of 1.20°, and the final values decreased to an average of 0.43°. This progress is shown in Figure 1, artificially increasing the mean. Without this progress, the group would have been very homogeneous.

Control group: The initial values had an average of 1.8°, while the final values reached an average of 0.77° (Figure 1).

Both groups enhanced their performance, but the improvement was more significant in the experimental group compared to the control group (0.77° vs. 0.41°), as highlighted in Table 1.

### 3.2. Stable Surface—Eyes Closed

Experimental group: The initial values measured for the 15 patients had an average of 1.25°, but the final values decreased to an average of 0.82°.

Control group: The initial values had an average of 1.22°, and the final values reached an average of 1.02°.

**Figure 2:**
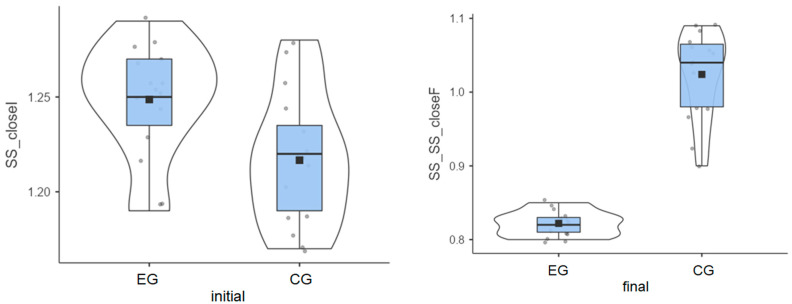
Stable surface distribution—eyes closed: (**left**) initial values and (**right**) final values for experimental group (EG) and control G = group (CG).

Although patients in the experimental group had a higher average value in the initial test, they achieved better results after the therapy sessions (Figure 2).

The experimental group had a greater decrease than the control group (0.43° vs. 0.19°) (Table 1), indicating better balance adaptation.

### 3.3. Unstable Surface—Eyes Open

**Figure 3:**
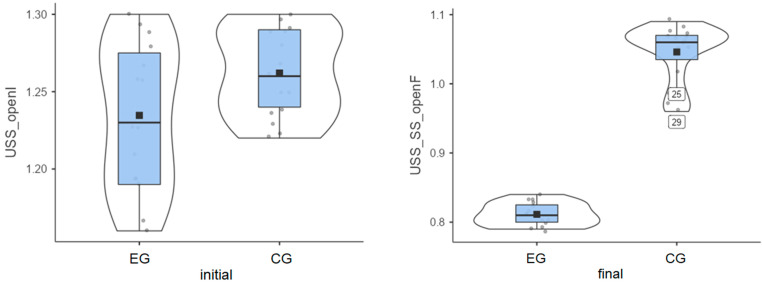
Unstable surface distribution—eyes open: (**left**) initial values and (**right**) final values for experimental group (EG) and control group (CG).

Experimental group: Patients started from an initial average value of 1.23° and reached a final average value of 0.81° (Figure 3). Their progress was satisfactory/very good, meaning that Biodex stimulates the balance between agonist and antagonist muscles, etc.

Control group: The initial average value was 1.26°, and the final average value was 1.05° (Figure 3), indicating some progress between the two testing sessions.

Again, the experimental group showed a stronger balance improvement (Table 1), with an average value of 0.42°, while the improvement obtained by the control group was 0.22°.

### 3.4. Unstable Surface—Eyes Closed (Most Challenging)

**Figure 4:**
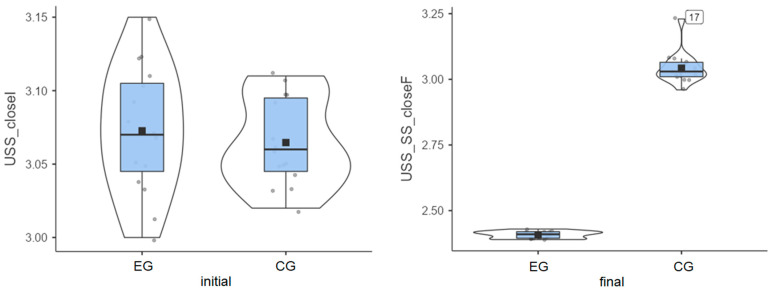
Unstable surface distribution—eyes closed: (**left**) initial values and (**right**) final values for experimental group (EG) and control group (CG).

Experimental group: Patients started from an initial average value of 3.07° and reached a final average value of 2.41° (Figure 4), thus exhibiting very good progress.

Control group: The initial average value of 3.06° was close to that of the experimental group, but progress was almost inexistent, as the final average value was 3.04° (Figure 4).

The experimental group showed a clear decrease in instability, while the control group recorded minor changes or even slight worsening in some cases (Table 1). The greatest improvement (0.66°) was achieved in the experimental group, while almost no improvement (0.02°) was recorded for the control group. The experimental group achieved the greatest improvement on the most challenging test.

The Shapiro–Wilk test was used to check the normal distribution of the variables. The results showed a normal distribution for the age of the participants (W = 0.97, *p* = 0.54), as well as for the following variables: SS_openI (W = 0.978, *p* = 0.767), SS_closedI (W = 0.979, *p* = 0.799), USS_openI (W = 0.96, *p* = 0.312), and USS_closedI (W = 0.977, *p* = 0.73). However, significantly different distributions from the normal were observed for SS_SS_openF (W = 0.901, *p* = 0.009), SS_SS_closedF (W = 0.925, *p* = 0.036), USS_SS_openF (W = 0.89, *p* = 0.005), and USS_SS_closedF (W = 0.754, *p* < 0.001). These results suggest that although most variables meet the assumption of normality, some post-intervention results show significant deviations from a normal distribution.

The initial measurements mostly follow a normal distribution. The final measurements for stable and unstable surfaces, especially under difficult conditions (eyes closed and unstable surfaces), show deviations from normality. This suggests that post-intervention balance performance may not follow a normal distribution, probably due to individual variability in response to training. For variables with a random distribution, nonparametric tests should be applied.

To test the homogeneity of variances between groups, Levene’s test was applied. The results indicate that the assumption of equality of variances was met for the participants’ age (F = 0.1096, *p* = 0.743), as well as for the variables SS_openI (F = 0.0859, *p* = 0.772), SS_closedI (F = 0.3949, *p* = 0.535), and USS_closedI (F = 1.6279, *p* = 0.212). In contrast, significant differences in variance between groups were found for the following variables: SS_SS_openF (F = 21. 7735, *p* < 0.001), SS_SS_closedF (F = 17.0097, *p* < 0.001), USS_openI (F = 6.543, *p* = 0.016), USS_SS_openF (F = 8.2882, *p* = 0.008), and USS_SS_closedF (F = 6.2878, *p* = 0.018). These results indicate a violation of the assumption of homogeneity of variances for some of the post-intervention scores and some baseline scores, suggesting the use of parametric tests for these variables with caution.

Most of the final measurements (especially on unstable surfaces) violate the assumption of equal variances, probably due to greater individual variability in response to the intervention. Standard parametric tests (e.g., *t*-tests, ANOVA) may not be suitable for these variables without adjustments. The variables highlighted in Table 2 do not meet the assumption of equal variances. If parametric tests are used, adjustments such as Welch’s *t*-test are needed.

### 3.5. Independent Samples t-Test Results

Table 2 highlights the results obtained for Student’s *t*-test and Welch’s *t*-test.

1. Age (*p* = 0.604): *p* > 0.05, so there is no significant age difference between groups, and Cohen’s d = 0.192, indicating a small effect size.
2. Stable Surface (SS)—Eyes Open Initial (*p* = 0.166): *p* > 0.05, so balance scores before training were similar, showing no significant difference. Cohen’s d = 0.52, indicating a medium effect size.
3. Stable Surface—Eyes Open Final (*p* < 0.001): *p* < 0.001, meaning that the experimental group showed much greater improvement, as the difference between groups is significant. Cohen’s d = −4.009, indicating a very large effect size.
4. Stable Surface—Eyes Closed Initial (*p* = 0.012): *p* < 0.05, meaning that the experimental group had better balance even before training. The difference is significant. Cohen’s d = 0.979, indicating a large effect size.
5. Stable Surface—Eyes Closed Final (*p* < 0.001): *p* < 0.001, so the experimental group improved significantly more. The difference is significant. Cohen’s d = −4.566, indicating a very large effect size.
6. Unstable Surface (USS)—Eyes Open Initial (*p* = 0.066): *p* > 0.05, meaning that both groups started at a similar level. The difference is not significant. Cohen’s d = −0.699, indicating a medium effect size.
7. Unstable Surface—Eyes Open Final (*p* < 0.001): *p* < 0.001, showing that the experimental group improved much more. Cohen’s d = −7.456, indicating an extremely large effect size.
8. Unstable Surface—Eyes Closed Initial (*p* = 0.562): *p* > 0.05, meaning that both groups started at a similar level. The difference is not significant. Cohen’s d = 0.214, indicating a small effect size.
9. Unstable Surface—Eyes Closed Final (*p* < 0.001): *p* < 0.001, showing that the experimental group improved much more. The difference is significant. Cohen’s d = −14.251, indicating a huge effect size.

Before training, the groups were mostly similar, except for SS_closedI (stable surface, eyes closed, initial testing) (*p* = 0.012), where the experimental group was slightly better. After training, the experimental group showed significantly greater improvements, especially in challenging conditions (unstable surface, eyes closed). Regarding effect size, Cohen’s d values indicate massive improvements in balance for the experimental group compared to the control group.

## 4. Discussion

The findings of this study emphasize the effectiveness of training using the BBS in improving coordination, proprioception, and both static and dynamic balance in individuals with T2DM and DPN. Notably, the most significant improvements were observed under challenging testing conditions, particularly on unstable surfaces and with eyes closed, indicating enhanced sensory integration and compensatory mechanisms in response to inadequate proprioceptive input.

Participants in the EG exhibited a mean improvement in postural stability of 0.43°, compared to 0.19° in the CG. This difference reflects superior postural adaptation and more efficient motor strategies. These enhancements are likely attributable to neuroplastic mechanisms, such as cortical reorganization in motor-related brain regions and improved cerebellar coordination. Repeated exposure to environments with diminished sensory input may promote sensory reweighting, allowing the central nervous system to better utilize visual and vestibular information when proprioception is impaired.

In conditions with a stable surface and eyes open, BBS training appears to facilitate effective multisensory integration—combining visual, vestibular, and proprioceptive input—at a level that exceeds what is typically achieved through conventional rehabilitation approaches. The structured, progressive, and feedback-driven nature of BBS intervention supports improved neuromuscular control, enhanced sensory integration, and greater postural stability, consistent with earlier research findings.

Under the stable surface with eyes closed condition, the EG demonstrated an improvement in sway angle from 1.25° to 0.82°, while the CG improved from 1.22° to 1.02°. Exercises in this setting promote proprioceptive and vestibular system adaptation and may support neuroplastic changes essential for postural control. Including such balance challenges in rehabilitation protocols is particularly valuable for reducing fall risk and improving mobility.

In the unstable surface with eyes open condition, participants trained under increased proprioceptive demands and dynamic postural control requirements, likely shifting reliance toward vestibular input and motor planning. This condition prompted patients to rely more heavily on visual and vestibular cues, thus enhancing adaptive responses to postural instability in dynamic environments.

The most demanding condition—unstable surface with eyes closed—posed the greatest sensory challenge and was associated with the most pronounced improvements. Training in this context promoted proprioceptive adaptation, enhanced vestibular response, activation of deep postural musculature, and cortical reorganization. These adaptations resulted in significant improvements in balance control and resilience to fall risk. Therefore, BBS training appears to be a valuable therapeutic intervention for improving functional mobility and overall safety in patients with DPN.

Descriptive statistical analysis revealed post-intervention improvements in all test conditions for both the EG and CG. However, the EG consistently achieved significantly greater gains, especially under high-demand sensory conditions. These outcomes support the superiority of technology-assisted interventions over traditional rehabilitation approaches in this clinical population.

These findings are aligned with the existing literature validating the use of the BBS in diabetic populations. Studies by Eftekhar-Sadat et al. (2015) and Daud et al. (2021) demonstrated significant improvements in balance metrics and reduced fall risk following BBS-based interventions [10,16]. Likewise, Stolarczyk et al. (2021) emphasized the importance of sensorimotor feedback and progressive proprioceptive loading in managing postural instability in patients with DPN [11]. The current study extends prior research by incorporating a structured 8-week intervention, offering more robust evidence for the medium-term efficacy of BBS training, particularly in populations at an elevated risk of functional decline.

Given the high prevalence of DPN and its strong association with falls, reduced mobility, and loss of independence, these findings carry significant clinical implications. The demonstrated ability of BBS training to improve proprioception and coordination—especially under the most demanding conditions—highlights its potential role as a core component in fall prevention strategies. Furthermore, the large effect sizes reported in this study point to meaningful clinical outcomes, not merely statistically significant changes. The neuroplastic benefits supported by BBS make it a compelling choice in rehabilitation programs aimed at improving multisensory integration and neuromuscular re-education.

Despite the promising outcomes, several limitations must be acknowledged. First, the relatively small sample size (n = 30) may limit statistical power and generalizability. Second, this study assessed outcomes over 8 weeks, so the long-term retention of balance improvements remains unclear. Third, this study did not directly assess real-life functional outcomes such as gait performance, number of falls, or daily activity levels. Additionally, blinding of participants or assessors was not explicitly described, which could introduce potential bias. Although some elements of structured intervention were included, this study does not meet the criteria for a randomized controlled trial as it lacked allocation concealment and blinding, and therefore, registration was not required under ICMJE guidelines. 

These factors should be considered when interpreting the results and in the design of future investigations. Future research should include larger, multicenter samples encompassing a wider range of demographic and clinical characteristics. Longitudinal studies spanning 6 to 12 months are needed to evaluate the persistence of training effects. Comparative studies assessing the efficacy of the BBS against other emerging rehabilitation modalities—such as virtual reality, vibration therapy, or wearable sensor-based systems—would be valuable. Additionally, research investigating underlying neurophysiological mechanisms (e.g., through electromyography or electroencephalography) may further clarify the biological basis of balance improvements. Incorporating patient-centered metrics such as quality-of-life indices and real-world functional data (e.g., fall diaries, activity trackers) would enhance clinical applicability. Moreover, evaluating the cost–benefit ratio of BBS implementation, particularly in resource-limited healthcare environments, is essential to support broader adoption.

While direct comparisons between the BBS and conventional rehabilitation methods remain limited, the present findings are consistent with those of other investigations [28,29,30,31,32,33]. For example, Eftekhar-Sadat et al. (2015) conducted a 6-month randomized trial involving 112 individuals aged 50–70 years with T2DM and peripheral neuropathy, confirming the value of BBS training for improving postural stability in older adults with diabetes [16]. Similar results were previously reported by Salsabili et al. (2011) [34], reinforcing the therapeutic relevance of the BBS in neuropathic population.

Conversely, the study by Ulbrich (2020) [33] reported no significant improvements in static or dynamic postural control following a 6-week training program, regardless of whether participants trained with eyes open or closed. However, individuals who trained with their eyes closed performed slightly better, suggesting a potential role for sensory deprivation in postural adaptation. Further studies are needed to determine whether eyes-closed protocols confer superior training effects [33].

Cox, Lephart, and Irrgang (1993) [35] compared the efficacy of balance training on stable versus unstable surfaces and found that although both groups improved, no significant difference emerged between the two conditions. Such results should be interpreted with caution due to limitations in the control of comorbidities or neurological disorders that may have influenced outcomes [35].

In a broader context, physical well-being is determined by a complex interplay of health status, physical activity, and nutrition, each contributing to optimal balance and functional capacit. Despite growing interest in digital rehabilitation technologies, the literature remains limited regarding the integration of variables such as exercise adherence, body perception, and the use of fitness tracking tools. This gap underscores the need for interdisciplinary research exploring how psychological, behavioral, and technological factors interact to support balance recovery and long-term functional outcomes in diabetic populations [36,37].

## 5. Conclusions

This quasi-experimental controlled study without random assignment of participants to groups demonstrated that an 8-week balance training program using the Biodex Balance System (BBS) led to significant improvements in postural stability and coordination in patients with T2DM and diabetic neuropathy. Compared to conventional rehabilitation, BBS-based training resulted in greater improvements across all conditions, especially under challenging balance scenarios (e.g., unstable surfaces with eyes closed), with large effect sizes indicating clinically meaningful benefits.

These findings support the integration of technology-assisted balance training into diabetes rehabilitation protocols, particularly for individuals at a high risk of falls due to sensory and motor deficits. The use of objective, feedback-driven systems like the BBS may enhance neuromuscular control and proprioceptive function more effectively than traditional approaches.

However, the results should be interpreted with caution given the limited sample size and short follow-up period. Future research should explore long-term outcomes, functional mobility in real-life settings, and cost-effectiveness to validate the utility of the BBS in broader clinical practice.

## Figures and Tables

**Table 1:** Descriptive statistics—clinical balance sensory integration test.

Descriptives	N		Mean				Standard Deviation		Minimum		Maximum		Shapiro–Wilk W		Shapiro–Wilk *p*	
Group	1	2	1	Dif. 1	2	Dif. 2	1.00	2.00	1	2	1	2	1	2	1	2
Age	15	15	60.3		59.7		3.28	2.97	55	55	66	64	0.975	0.95	0.923	0.5
SS_openI	15	15	1.2	0.77	1.18	0.18	0.04	0.04	1.13	1.1	1.27	1.3	0.980	0.92	0.969	0.22
SS_openF	15	15	0.43		0.77		0.02	0.12	0.4	0.6	0.46	1	0.901	0.90	0.099	0.08
SS_closedI	15	15	1.25	0.43	1.22	0.20	0.03	0.03	1.19	1.17	1.29	1.28	0.92	0.95	0.180	0.49
SS_closedF	15	15	0.82		1.02		0.02	0.06	0.8	0.9	0.85	1.1	0.919	0.89	0.183	0.06
USS_openI	15	15	1.23	0.42	1.26	0.21	0.05	0.03	1.16	1.2	1.3	1.3	0.923	0.92	0.214	0.16
USS_openF	15	15	0.81		1.05		0.02	0.04	0.79	1	0.84	1.1	0.924	0.82	0.223	0.01
USS_closedI	15	15	3.07	0.66	3.06	0.02	0.04	0.03	3	3	3.15	3.1	0.98	0.92	0.972	0.18
USS_closedF	15	15	2.41		3.04		0.01	0.06	2.39	3	2.43	3.2	0.877	0.8	0.042	0.00

**Table 2:** Independent samples *t*-Test.

		Statistic	df	*p*	Mean diff.	SE diff.	Significant Difference	Effect Size (Cohen’s d)
**Age**	Student’s t	0.525	28	0.604	0.60	1.14		0.19
	Welch’s t	0.525	27.7	0.604	0.60	1.14		0.19
**SS_openI**	Student’s t	1.424	28	0.166	0.02	0.02		0.52
	Welch’s t	1.424	28	0.166	0.02	0.02		0.52
**SS_SS_openF**	Student’s t	−10.98	28	<0.001	−0.34	0.03		−4.01
	Welch’s t	−10.98	14.9	**<0.001**	−0.34	0.03	Yes	−4.01
**SS_closedI**	Student’s t	2.681	28	**0.012**	0.03	0.01	Yes	0.98
	Welch’s t	2.681	27.5	0.012	0.03	0.01		0.98
**SS_SS_closedF**	Student’s t	−12.505	28	<0.001	−0.20	0.02		−4.57
	Welch’s t	−12.505	16.2	**<0.001**	−0.20	0.02	Yes	−4.57
**USS_openI**	Student’s t	−1.914	28	0.066	−0.03	0.01		−0.70
	Welch’s t	−1.914	23	0.068	−0.03	0.01		−0.70
**USS_SS_openF**	Student’s t	−20.42	28	<0.001	−0.23	0.01		−7.46
	Welch’s t	−20.42	18.3	**<0.001**	−0.23	0.01	Yes	−7.46
**USS_closedI**	Student’s t	0.587	28	0.562	0.01	0.01		0.21
	Welch’s t	0.587	25.2	0.563	0.01	0.01		0.21
**USS_SS_closedF**	Student’s t	−39.028	28	<0.001	−0.63	0.02		−14.25
	Welch’s t	−39.028	15.4	<0.001	−0.63	0.02	Yes	−14.25

*Note.* H_a_ μ _1_ ≠ μ _2_; Levene’s test is significant (*p* < 0.05), suggesting a violation of the assumption of equal variances.

## Data Availability

The data are contained within the main text of the article.

## Abbreviations

The following abbreviations are used in this manuscript:

T2DM	type 2 diabetes mellitus
DPN	diabetic peripheral neuropathy
BBS	Biodex Balance System
SOT	sensory organization test
pLOS	mean peak sway amplitude
SMMSE	Standardized Mini-Mental Scale
GSI	general stability index
FPI	frontal–posterior index
MLI	medial–lateral index
